# RNA-Sequencing Analysis of Paternal Low-Protein Diet-Induced Gene Expression Change in Mouse Offspring Adipocytes

**DOI:** 10.1534/g3.119.400181

**Published:** 2019-05-03

**Authors:** Nhung Hong Ly, Toshio Maekawa, Keisuke Yoshida, Yang Liu, Masafumi Muratani, Shunsuke Ishii

**Affiliations:** * RIKEN Cluster for Pioneering Research, 3-1-1 Koyadai, Tsukuba, Ibaraki 305-0074, Japan; † Department of Functional Genomics, Graduate School of Comprehensive Human Sciences, University of Tsukuba, Tsukuba, Ibaraki 305-8577, Japan; ‡ Department of Genome Biology, Faculty of Medicine, University of Tsukuba, Tsukuba, Ibaraki 305-8577, Japan

**Keywords:** Paternal diet, Low-protein diet, Adipocytes, ChREBP

## Abstract

Increasing evidence indicates that parental diet affects the metabolism and health of offspring. It is reported that paternal low-protein diet (pLPD) induces glucose intolerance and the expression of genes involved in cholesterol biosynthesis in mouse offspring liver. The aim of the present study was to determine the effect of a pLPD on gene expression in offspring white adipose tissue (WAT), another important tissue for the regulation of metabolism. RNA-seq analysis indicated that pLPD up- and down-regulated 54 and 274 genes, respectively, in offspring WAT. The mRNA expression of many genes involved in lipogenesis was down-regulated by pLPD feeding, which may contribute to metabolic disorder. The expression of carbohydrate response element-binding protein β (*ChREBP*-*β*), an important lipogenic transcription factor, was also significantly lower in the WAT of pLPD offspring, which may have mediated the down-regulation of the lipogenic genes. By contrast, the LPD did not affect the expression of lipogenic genes in the WAT of the male progenitor, but increased the expression of lipid oxidation genes, suggesting that a LPD may reduce lipogenesis using different mechanisms in parents and offspring. These findings add to our understanding of how paternal diet can regulate metabolism in their offspring.

Maternal undernutrition increases the risk of lifestyle-related diseases, such as cardiovascular disease and type II diabetes, in children (Black *et al.* 2008), as originally reported by Barker *et al.* (Barker *et al.* 1993) and also documented in the case of the Dutch famine (de Rooij *et al.* 2006). Studies using mice have demonstrated that *in utero* undernutrition of F1 embryos alters the germline DNA methylome of F1 adult males and induces glucose intolerance in the F2 generation through the paternal line (Radford *et al.* 2014). Thus, although the effect of the maternal environment has been thoroughly investigated, an increasing number of studies also indicate that there are associations between paternal nutrition and offspring health. In addition, an epidemiological study conducted in Sweden showed that grandpaternal famine is associated with obesity and cardiovascular disease in grandchildren (Kaati *et al.* 2002).

A number of mouse models have demonstrated the effects of paternal dietary manipulation on offspring metabolism and health. High-fat diet feeding of male rats induces pancreatic β cell dysfunction and glucose intolerance in female offspring (Ng *et al.* 2010). In addition, the offspring of male mice fed a low-protein diet (LPD) exhibit higher expression of genes involved in lipid and cholesterol biosynthesis in neonatal liver (Carone *et al.* 2010). This paternal LPD (pLPD) also induces greater adiposity, glucose intolerance, and cardiovascular dysfunction, and increases serum tumor necrosis factor α concentrations, in offspring mice (Watkins and Sinclair 2014). Finally, fasting of male mice before mating leads to changes in serum glucose, insulin-like growth factor 1 (IGF1), and corticosterone concentrations in their offspring (Anderson *et al.* 2006). Although these studies demonstrate that paternal undernutrition transgenerationally induces metabolic disorders, the tissue-specific effects on gene expression in the offspring have not been evaluated. Therefore, the aim of the current study was to analyze the effect of a pLPD on gene expression in offspring adipocytes, which contribute to whole-body metabolic regulation.

Lipid metabolism in white adipose tissue (WAT) plays an important role in metabolic homeostasis (Rosen and Spiegelman 2006; Virtue and Vidal-Puig 2010). *De novo* lipogenesis in WAT, which converts non-lipid precursors to fatty acids, is down-regulated in obesity, and the reinstatement of lipogenesis in WAT prevents obesity-dependent insulin resistance (Cao *et al.* 2008; Huo *et al.* 2012). Palmitoleate, a fatty acid produced by lipogenesis, appears to mediate the insulin resistance caused by the lower lipogenic rate in WAT (Yang *et al.* 2011; Huo *et al.* 2012). The transcription factor carbohydrate response element-binding protein (ChREBP, also known as MLXIPL) is an important regulator of lipogenesis in WAT (Uyeda & Repa 2006; Herman *et al.* 2012; Baraille *et al.* 2015). It is a member of the basic-helix-loop-helix leucine zipper transcription factor family, and binds to glucose or carbohydrate response elements (ChOREs), which are composed of two E-boxes (CACGTG) or E-box-like sequences, after heterodimerizing with max-like protein X (MLX) (Yamashita *et al.* 2001; Ma *et al.* 2006). Two isoforms of ChREBP, ChREBP-α and the N-terminally truncated ChREBP-β, which is expressed from an alternative promoter, have been identified (Herman *et al.* 2012). In response to high concentrations of glucose in WAT, phosphatase 2A is activated by metabolites and dephosphorylates ChREBP-α, leading to the translocation of ChREBP-α into the nucleus and the induction of *ChREBP*-*β* gene transcription following binding of ChREBP-α to the upstream promoter (Kabashima *et al.* 2003; Herman *et al.* 2012; Baraille *et al.* 2015; Abdul-Wahed *et al.* 2017). ChREBP-β more potently induces the expression of lipogenic genes than the more abundant ChREBP-α (Herman *et al.* 2012). Furthermore, adipose-specific ChREBP knockout mice exhibit systemic insulin resistance (Vijayakumar *et al.* 2017). In contrast to the situation in WAT, lipogenesis in the liver is enhanced in obesity, which also promotes insulin resistance and lipotoxicity (Beaven *et al.* 2013). This may be, at least in part, due to greater synthesis of ceramides from palmitate, which potently induce insulin resistance (Chavez & Summers 2012), and activation of the innate immune system by saturated fatty acids (Fessler *et al.* 2009). Thus, lipogenesis in the WAT and liver has opposite effects on insulin sensitivity (Eissing *et al.* 2013).

To determine the mechanism underlying the pLPD-induced metabolic disorder in mouse offspring, we investigated the effect of pLPD on gene expression in the WAT of these animals. We found that pLPD caused lower expression of a number of genes involved in lipogenesis. In particular, *ChREBP*-*β* expression was lower, whereas insulin-like growth factor-binding protein 2 (*Igfbp2*) expression was higher, suggesting that an impairment in insulin signaling induced by IGFBP2 may suppress ChREBP-α-dependent *ChREBP*-*β* expression. By contrast, LPD did not affect the expression of lipogenic genes in WAT of the male progenitor, but increased the expression of lipid oxidation genes, suggesting that LPD may reduce lipid storage by activating lipid oxidation.

## Materials and Methods

### Mice and diets

All animal care and experiments were conducted in accordance with the guidelines of the Animal Care and Use Committee of the RIKEN Institute. C57BL/6 mice (C57BL6/JJmsSlc background) were obtained from Japan SLC (Shizuoka, Japan), and diets were obtained from Oriental Yeast Co (Shiga, Japan). To compare gene expression in inguinal WAT (iWAT) in the offspring of mice fed a control diet (CD) or a pLPD with that previously reported in liver (Carone *et al.* 2010), all procedures and the composition of the diet were matched to those described by Carone *et al.* (Carone *et al.* 2010). Mice were fed with the CD for two generations to minimize the transgenerational effect of the chow fed by the animal supplier. After weaning at 3 weeks of age, male mice were fed with the CD or LPD, while female mice were fed with the CD. At 10–12 weeks of age, male and virgin female mice were placed in the same cage for mating, in which they consumed CD, for 2 days only, to avoid a significant reduction in the effect of the LPD in male mice. After this, the males were removed and the pregnant females were fed the CD until their offspring reached 3 weeks of age, when they were weaned. From this time, the offspring were fed the CD and killed at 5 weeks of age, when their iWAT was rapidly dissected and flash-frozen in liquid N_2_.

### RNA sequencing (RNA-seq) analysis

Total RNA was isolated from iWAT using TRIZOL reagent (Thermo Fisher Scientific, Waltham, MA). RNA quality was evaluated using an RNA 6000 Pico kit (Agilent, Santa Clara, CA). Fifty nanograms of total RNA was used for RNA sequencing (RNA-seq) library preparation with a NEBNext rRNA Depletion Kit and a NEBNext Ultra Directional RNA Library Preparation Kit (New England Biolabs, Ipswich, MA), then 2 × 36 base pair-end sequencing was performed using NextSeq500 (Illumina, San Diego, CA) by Tsukuba i-Laboratory LLP (Tsukuba, Ibaraki, Japan).

### Bioinformatics and statistics for RNA-seq data

RNA-seq data were analyzed using CLC Genomics Workbench version 9.5.1 (QIAGEN) (Nielsen *et al.* 2010). Sequences were mapped to the mouse mm10 genome. Expression values were calculated as “reads per kilobase per million reads” using CLC Genomics Workbench (Qiagen). Genes were considered to be differentially expressed when false discovery rate (FDR) adjusted *P*-values were < 0.05. Pathway enrichment analysis for differentially expressed genes (DEGs) was conducted using the Kyoto Encyclopedia of Genes and Genomes (KEGG) database (Kanehisa *et al.* 2014) and the Database for Annotation, Visualization, and Integrated Discovery (DAVID) (Huang da *et al.* 2009a; Huang da *et al.* 2009b).

### Quantitative RT-PCR (qRT-PCR) analysis

RT-PCR analysis was performed using the OneStep SYBR Green PCR mix (Takara, Shiga, Japan), following the manufacturer’s instructions. The quantitative RT-PCR (qRT-PCR) was performed using a 7500 Fast Real-time PCR System (Applied Biosystems, Foster City, CA). Oligonucleotide primers were designed to produce 70–150 base amplification products. The primers used are shown in Table S1. *36B4*, which encodes Rplp0 ribosomal protein, large, P0, was used as a reference gene, and the relative expression levels of each target gene were quantified using the 2^−ΔΔCt^ method (Livak and Schmittgen 2001).

### Quantitative chromatin immunoprecipitation (qChIP)

Chromatin immunoprecipitation (ChIP) assays were performed as described previously, with some modifications (Haim *et al.* 2013; Yoshida *et al.* 2015). Fresh iWAT from 5-week-old offspring was rinsed once with 1× phosphate-buffered saline (PBS) supplemented with protease inhibitors (PI), then minced into small pieces. Minced tissues were fixed with 1% formaldehyde for 20 min at room temperature with moderate shaking, then washed twice to remove lipid. Small adipose tissue pieces were resuspended in 1 ml of adipocyte lysis buffer 1 (50 mM HEPES, pH 7.5, 140 mM NaCl, 1 mM EDTA, 10% glycerol, 1% NP-40, and 0.25% Triton X-100) supplemented with PI, rotated for 5 min, then homogenized using a Dounce homogenizer (30 strokes). Samples were rotated at 4° for 15 min (vortexed every 5 min), then 20 additional strokes were performed. The samples were centrifuged (5 min, 3,000 rpm, 4°), and the pellet was washed three times in lysis buffer 2 (10 mM Tris-HCl, pH 8.0, 200 mM NaCl, 0.5 mM EGTA, 1 mM EDTA, and PI) to remove lipid. For DNA shearing, the nuclear pellet was resuspended in 200 µl of sodium dodecyl sulfate (SDS) lysis buffer (1% SDS, 10 mM EDTA, pH 8.1, with 50 mM Tris-HCl, pH 8.1) supplemented with PI, and incubated on ice for 20 min prior to sonication. Sonication was optimized for a DNA fragment size of ∼200–600 bases. After sonication, lysis buffer 3 (10 mM Tris-HCl, pH 8.0, 100 mM NaCl, 0.5 mM EGTA, 1 mM EDTA, 1% Triton X-100, 0.1% sodium deoxycholate, and PI) was added, and the final SDS concentration was adjusted to 0.1%. Dynabead Protein A was prepared by washing twice with PBS containing bovine serum albumin. ChREBP antibody (NBP2-44307, Novus, Littleton, CO) and rabbit IgG control (sc-2017, Santa Cruz, Santa Cruz, CA) were bound to the beads by incubation for 3 h at 4°, then the beads were washed three times. A mixture of beads and lysate was rotated for 14 h at 4°. Next, the beads were washed six times, before elution in elution buffer. The eluted samples were reverse-crosslinked overnight at 65° and then treated with RNase and Protease K at 37°. Finally, DNA was purified using a QIAgen Kit prior to qRT-PCR. The ChIP-qPCR primers used are listed in Table S1.

### Statistics for non-RNA-seq data

Data are presented as mean ± SD, and group comparisons were performed using independent sample *t*-tests. The significance level was set at *P* < 0.05.

### Data availability

Figure S1 contains the volcano plot and GO analysis for RNA-seq data of F0 generation under LPD. Table S1 contains the information on primer sequences used for qRT-PCR and qChIP. File S1 contains the list of up- and down-regulated genes induce by pLPD in male offspring iWAT. File S2 contains the list of up- and down-regulated genes induce by LPD in father iWAT. The raw data and processed data files were deposited in the gene expression omnibus (GEO) database of NCBI with the accession number GSE121774. Supplemental material available at FigShare: https://doi.org/10.25387/g3.7996325.

## Results

### Down-regulation of lipogenic gene expression in the iWAT of the offspring of mice fed a pLPD

**Figure 1:**
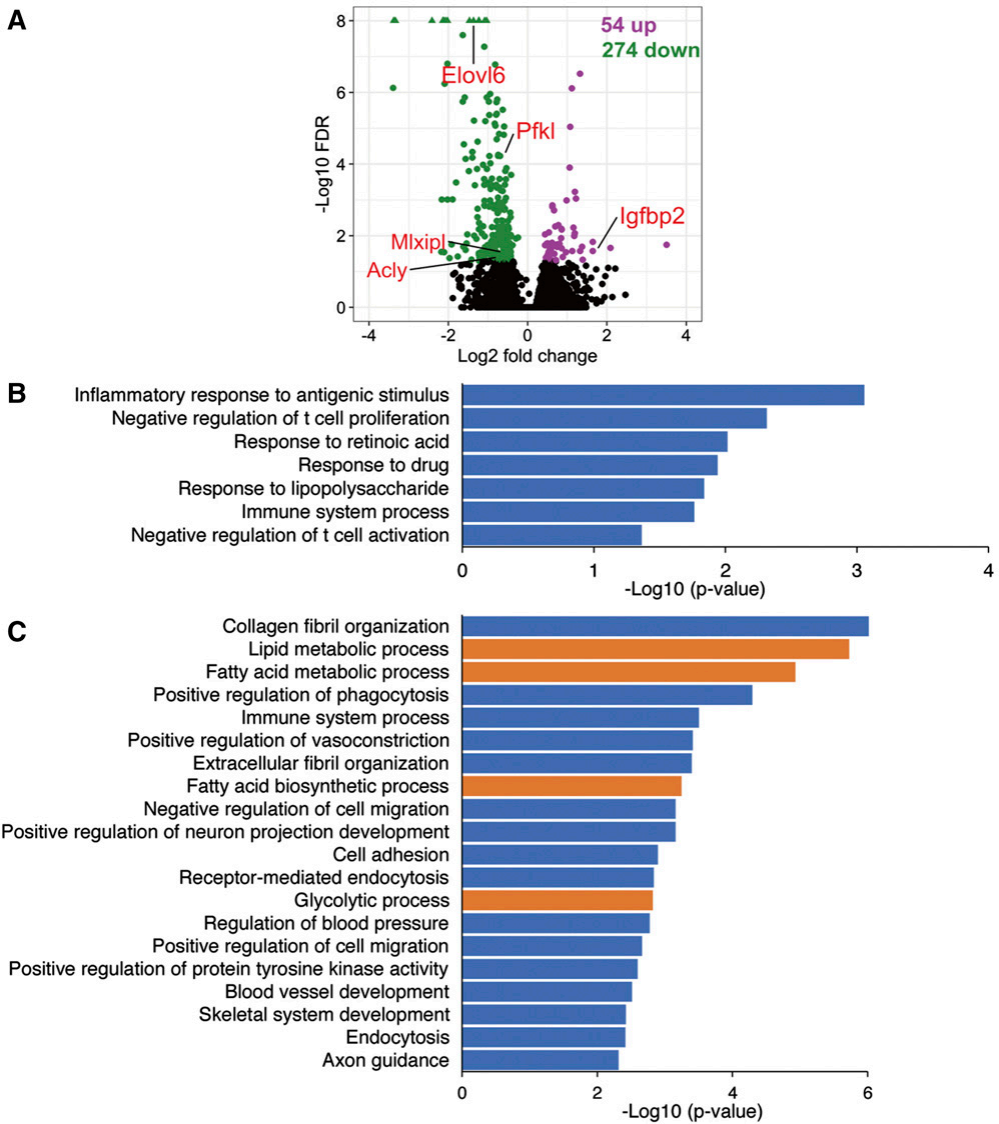
The effects of a pLPD on gene expression in offspring iWAT. (A) F0 male mice (n = 3) were fed with a CD or a LPD and mated with female mice fed a CD to generate F1 offspring. Using total RNA extracted from the inguinal white adipose tissue (iWAT) of F1 male offspring (three mice with three different mothers), gene expression was analyzed using RNA-seq. A volcano plot depicting the differentially expressed genes (n = 328) induced by pLPD feeding in male offspring iWAT. pLPD up-regulated 54 genes (purple spots) and down-regulated 274 genes (green spots) (FDR < 0.05). Four representative down-regulated lipogenic genes are shown. (B and C) Gene ontology analysis for the biological processes (GO-BP) represented among the up- and down-regulated genes, respectively. The *P*-value for the enrichment was obtained using a modified Fisher’s exact test. Y-axis: name of the BP term; x-axis: −log10 (*P*-value). Seven GO terms (*P* < 0.05) were over-represented among the up-regulated genes and are mainly associated with the immune response (B). For the down-regulated genes (FDR < 0.05), only the top 20 GO-BP terms are shown. The down-regulated genes included many GO-BP terms involved in metabolism, such as lipid metabolism and glycolysis (orange bar) (C).

To determine the effects of a pLPD on gene expression in offspring iWAT, we used essentially the same feeding regimen reported by Carone *et al.* (Carone *et al.* 2010). Mice were first fed with the CD for two generations to minimize the transgenerational effect of the chow provided by the animal supplier. After weaning at 3 weeks of age, male F0 mice were fed the CD or LPD, while female F0 mice were fed the CD. Mating was performed within a 2 day period to avoid any significant reduction in the effects of the LPD on male F0 mice. The male F1 offspring obtained were killed at 5 weeks of age, and total RNA was prepared from their iWAT, which was used for transcriptomic analysis by RNA-seq. This analysis identified 54 genes that were up- and 274 genes that were down-regulated in iWAT from mice that were the offspring of male progenitors fed with the LPD compared with those from CD fathers (Figure 1A).

To identify the pathways associated with the DEGs, lists of up- and down-regulated genes were analyzed for KEGG pathway enrichment using the DAVID database. Seven biological processes (BPs) were significantly over-represented among the up-regulated genes, which were mainly involved in immunity, such as T cell proliferation and activation, immune system processes, and the response to lipopolysaccharide (Figure 1B and File S1). The down-regulated genes contained numerous representatives from metabolic pathways, such as lipid metabolism, fatty acid biosynthesis, and glycolysis. Another major enriched group is involved in adipocyte development and function, including collagen fibril organization, positive regulation of phagocytosis, extracellular fibril organization, and related annotations (Figure 1C and File S1). Thus, the results of genome-wide profiling indicate metabolic and developmental programming of the WAT of male offspring in response to a pLPD.

**Figure 2:**
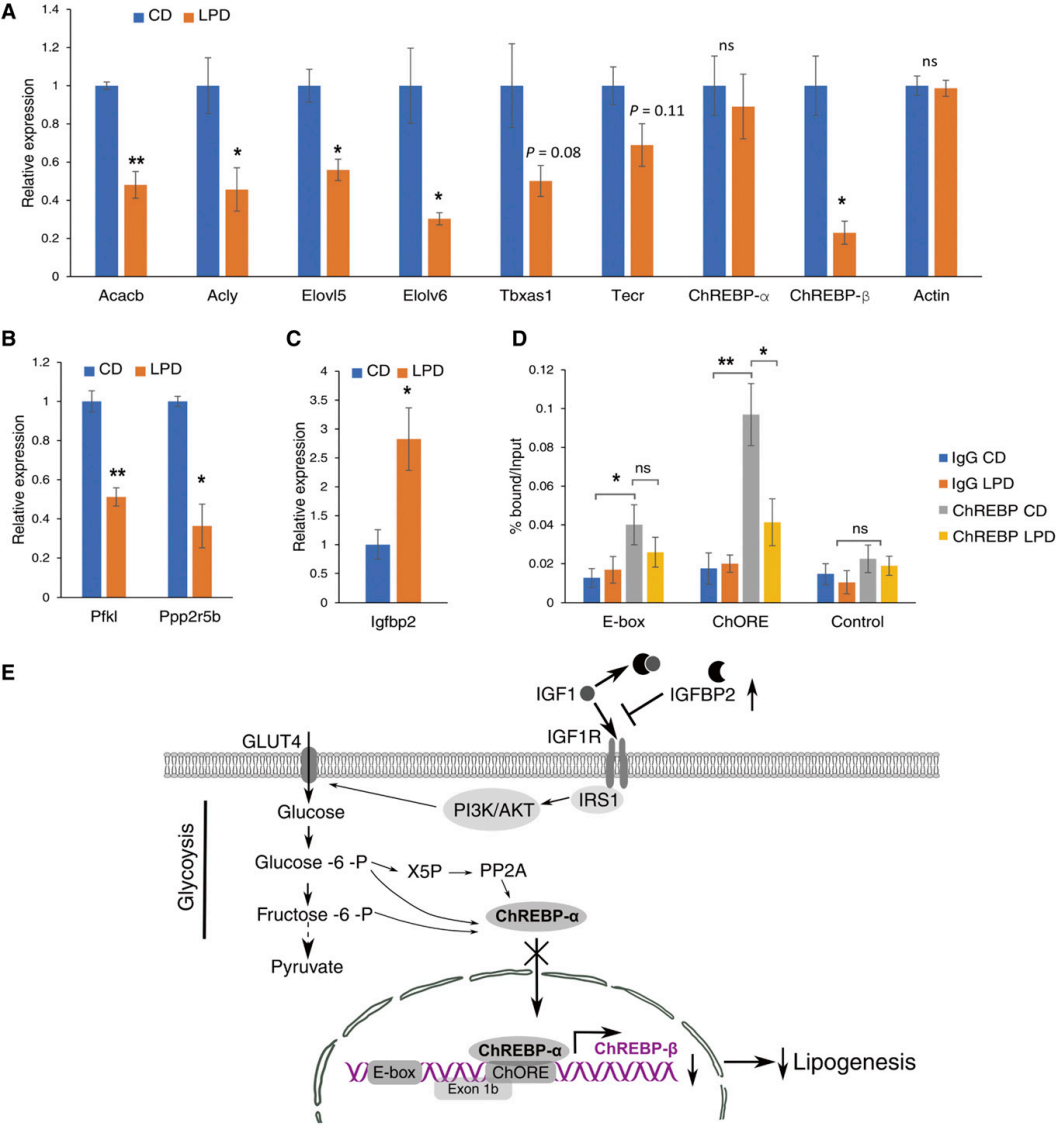
pLPD-induced down-regulation of lipogenic genes in F1 offspring iWAT. (A) Differential mRNA expression of selected lipogenic genes shown in Figure 1A was corroborated using qRT-PCR (n = 3). Gene expression was normalized to the reference gene *36B4*, and actin was used as a negative control. Bars represent the mean ± SD *, *P* < 0.05; **, *P* < 0.01; ns, not significant; CD, control diet; LPD, low-protein diet. (B and C) The down-regulation of *Pfkl* and *Ppp2r5b*, and up-regulation of *Igfbp2*, in F1 iWAT was confirmed by qRT-PCR. (D) pLPD reduced the amount of ChREBP bound to the *ChREBP*-*β* gene promoter in F1 offspring iWAT, demonstrated using qChIP of F1 offspring iWAT and an anti-ChREBP antibody. Primers that amplified the region containing the *ChREBP*-*β* gene promoter were used for qPCR. Mean values relative to the input ± SD are shown (n = 3). *, *P* < 0.05; **, *P* < 0.01; ns, not significant. (E) A speculative scheme for the down-regulation of the lipogenic genes in F1 iWAT.

Because lipogenesis in WAT is down-regulated in obesity and the reinstatement of lipogenesis in WAT prevents obesity-dependent insulin resistance (Cao *et al.* 2008; Huo *et al.* 2012), we focused on the genes involved in lipogenesis. The genes that were down-regulated by a pLPD were significantly enriched for BPs related to lipid biosynthesis (Figure 1C). Many genes enriched in this pathway are key enzymes responsible for the regulation of triglyceride biosynthesis, including ATP citrate lyase (*Acly*), acetyl-CoA carboxylase 2 (*Acacb*), elongation of very-long-chain fatty acids protein 5 (*Elovl5*), elongation of very-long-chain fatty acids protein 6 (*Elovl6*), thromboxane A synthase 1 (*Tbxas1*), and *trans*-2, 3-enoyl-CoA reductase (*Tecr*). To attempt to corroborate the RNA-seq data, we measured the relative expression levels of these genes by qRT-PCR, using *36B4* as a reference gene. The results were consistent with the RNA-seq data and indicated that the expression levels of lipogenic genes were significantly reduced by pLPD feeding (Figure 2A). In addition, the mRNA expression of *ChREBP*, which up-regulates the expression of multiple lipogenic genes (Uyeda & Repa 2006; Herman *et al.* 2012; Baraille *et al.* 2015), was also was also significantly reduced by pLPD feeding (log_2_FC = −1.48, FDR = 0.03) (Figure 1A). We examined the expression of the two ChREBP isoforms by qRT-PCR. Interestingly, expression of ChREBP-β, which more potently induces lipogenesis genes compared to more abundant ChREBP-α, was markedly reduced in LPD offspring, whereas, *ChREBP*-*α* expression remained unchanged (Figure 2A).

### Possible mechanism underlying the pLPD-induced down-regulation of lipogenic genes

It was previously shown that glycolysis is tightly linked to the transcription of the *ChREBP*-*β* gene from the upstream promoter following dephosphorylation of ChREBP-α, which leads to its nuclear translocation (Kabashima *et al.* 2003; Uyeda & Repa 2006; Herman *et al.* 2012; Baraille *et al.* 2015). In offspring WAT, mRNA expression of phosphofructokinase (*Pfkl*), one of the key glycolytic enzymes, and protein phosphatase 2 regulatory subunit B’β (*Ppp2rb5*), which dephosphorylates ChREBP-α (Uyeda & Repa 2006; Kabashima *et al.* 2003), was also reduced by pLPD feeding (Figure 2B). These results are consistent with pLPD reducing glycolytic rate and the dephosphorylation of ChREBP-α, which would suppress *ChREBP*-*β* gene transcription in offspring iWAT.

This finding prompted us to look for altered expression of upstream regulators of glycolysis. Interestingly, we found that the mRNA expression of *Igfbp2*, a regulator of IGF1 signaling, which affects glucose metabolism, was up-regulated by pLPD feeding (Figure 2C). IGF1 signaling substantially overlaps with insulin signaling, sharing intermediates such as insulin receptor substrate 1, and the phosphoinositide 3-kinase/AKT pathway, activation of which increases glucose uptake by up-regulating the expression of glucose transporter 4, regulating its intracellular trafficking, and by stimulating the activity of some glycolytic enzymes (Ward and Thompson 2012). IGFBP2 inhibits IGF1 activity by binding to the IGF1 receptor and preventing access of IGF1, resulting in the suppression of IGF1 signaling glycolytic enzymes (Ward and Thompson 2012). Therefore, up-regulation of IGFBP2 may inhibit glycolysis, leading to suppression of the nuclear translocation of ChREBP-α.

Previous studies have identified two putative ChOREs, the first of which is an E-box region upstream of exon 1b, and the second of which is downstream of exon 1b (Herman *et al.* 2012; Zhang *et al.* 2015). We therefore performed a ChIP-qPCR experiment using an anti-ChREBP antibody that recognizes both ChREBP isoforms to analyze the binding of ChREBP to these two sites. More ChREBP was found to bind to the second putative ChORE, but the amount bound was 60% lower in F1 mice with a pLPD-fed parent than in those with a CD-fed parent (Figure 2D). Thus, pLPD reduced the amount of ChREBP, presumably ChREBP-α, binding to the promoter of *ChREBP*-*β*, leading to a reduction in the expression of *ChREBP*-*β*. Taken together, these data suggest that down-regulation of lipogenic genes can be explained, at least in part, by an increase in *Igfbp2* expression, which suppresses IGF1 signaling, *ChREBP*-*β* expression, and therefore lipogenic gene expression (Figure 2E).

### Comparison of gene expression patterns in iWAT From F1 offspring and male progenitors

**Figure 3:**
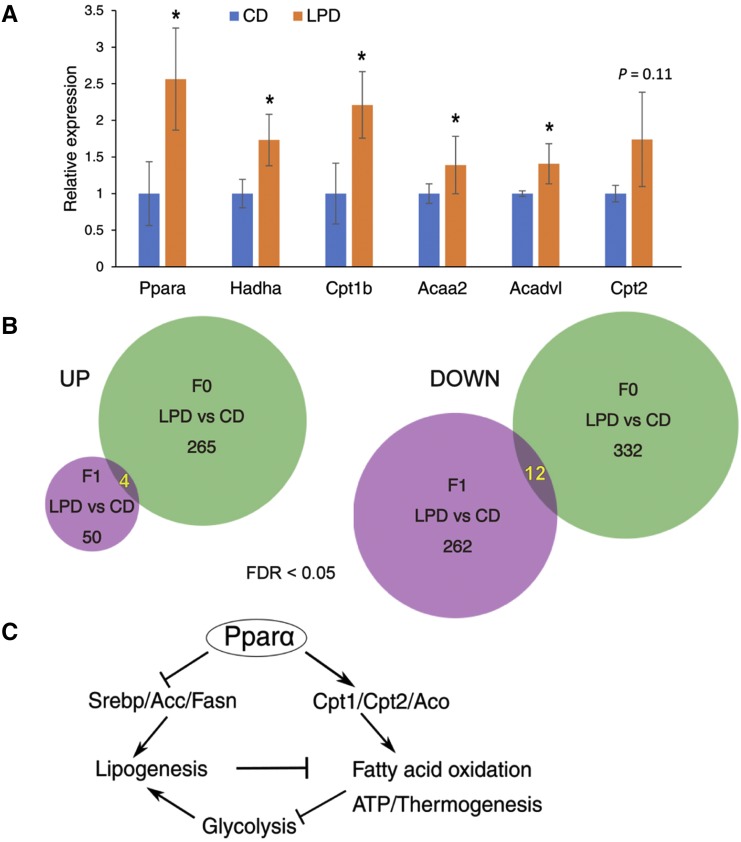
Comparison of the gene expression changes induced by LPD feeding in F0 male progenitor and F1 offspring iWAT. (A) F0 male mice were fed a CD or a LPD, then total RNA was prepared from the iWAT of F0 mice at 10–12 weeks of age, and used for RNA-seq analysis. The results shown in Figure S1 suggested the down-regulation of genes involved in fatty acid oxidation. The down-regulation of key genes involved in fatty acid oxidation was confirmed by qRT-PCR (n = 4). Target gene expression was normalized to that of the reference gene *36B4*. *, *P* < 0.05; ns, not significant. Bars represent the mean ± SD (B) A comparison of the genes affected by LPD in male F0 and F1 offspring iWAT. The Venn diagram shows the numbers of genes that were up- or down-regulated by LPD feeding in male F0 and F1 offspring iWAT. CD, control diet; LPD, low-protein diet. (C) A speculative schematic view of the effect of a LPD on metabolism in F0 iWAT.

We next asked whether the alteration in gene expression pattern in F1 offspring iWAT mimicked that in their male parents. For this purpose, we performed transcriptome profiling of F0 iWAT in 10–12-week-old male mice fed the LPD or CD. RNA-seq analysis identified 269 genes up- and 334 genes down-regulated in F0 iWAT from mice fed the LPD compared with those fed the CD (Figure S1A). The pathways associated with DEGs were analyzed for KEGG pathway enrichment using the DAVID database. The up-regulated genes were significantly enriched for genes involved in lipid metabolism, oxidation-reduction processes, and the tricarboxylic acid (TCA) cycle, whereas the down-regulated genes are involved in muscle and cardiac function and development (Figure S1B and S1C, and File S2). Many genes implicated in lipid metabolism were induced in F0 iWAT by the LPD, including peroxisome proliferator-activated receptor α (*Ppara*), carnitine palmitoyltransferase 1B (*Cpt1b*), carnitine palmitoyltransferase II (*Cpt2*), hydroxyacyl-CoA dehydrogenase trifunctional multienzyme complex subunit α (*Hadha*), acetyl-CoA acyltransferase 2 (*Acaa2*), and acyl-CoA dehydrogenase very-long-chain (*Acadvl*). The results of qRT-PCR indicated that the mRNA expression of *Ppara*, *Hadha*, *Cpt1b*, *Acaa2*, and *Acadvl* was significantly higher in LPD-fed mouse iWAT, and that of *Cpt2* tended to be higher (Figure 3A). Because PPARα is a transcription factor that up-regulates the transcription of multiple genes involved in lipid oxidation (Leone *et al.* 1999), higher expression of PPARα may cause higher expression of lipid oxidation genes.

Next, we compared the overlap between sets of DEGs in F1 and F0 iWAT. Among the 54 up-regulated genes in the F1 mice and the 269 up-regulated genes in the F0 mice, there were only four common genes (Figure 3B). Furthermore, only 12 genes showed overlapping differential expression among the 274 and 334 down-regulated genes in the F1 and F0 iWAT, respectively. These results indicate that the transcriptomic changes in offspring iWAT were dissimilar from those in F0 iWAT. Analysis of the DEGs suggested that lipogenesis is down-regulated in F1 iWAT, whereas lipolysis is up-regulated in F0 iWAT. Lipogenesis and lipolysis are both tightly regulated processes, and they work oppositely to accumulate lipid. Among the genes that were up-regulated in the F0 males, PPARα is a key regulator of fatty acid oxidation, which targets many genes in this pathway, including *Cpt1*, *Cpt2*, and *Aco* (Figure 3C). PPARα also can inhibit lipogenesis by suppressing *Srebp*, *Acc*, and *Scd* expression or by reducing the expression of glycolytic genes (Finck and Kelly 2002). The up-regulation of fatty acid oxidation is usually associated with greater energy production and thermogenesis. Consistent with this, genes involved in the TCA cycle and uncoupling protein 1 (*Ucp1*) were up-regulated by LPD feeding, according to the RNA-seq data (Figure S1B, File S2) (Figure 3C). Thus, in contrast to the reduction in lipogenesis in offspring iWAT, mediated through altered ChREBP expression, lipogenesis may be down-regulated in F0 iWAT secondary to enhanced lipolysis, mediated through PPARα.

### Comparison of the pLPD-regulated genes in offspring iWAT and liver

**Figure 4:**
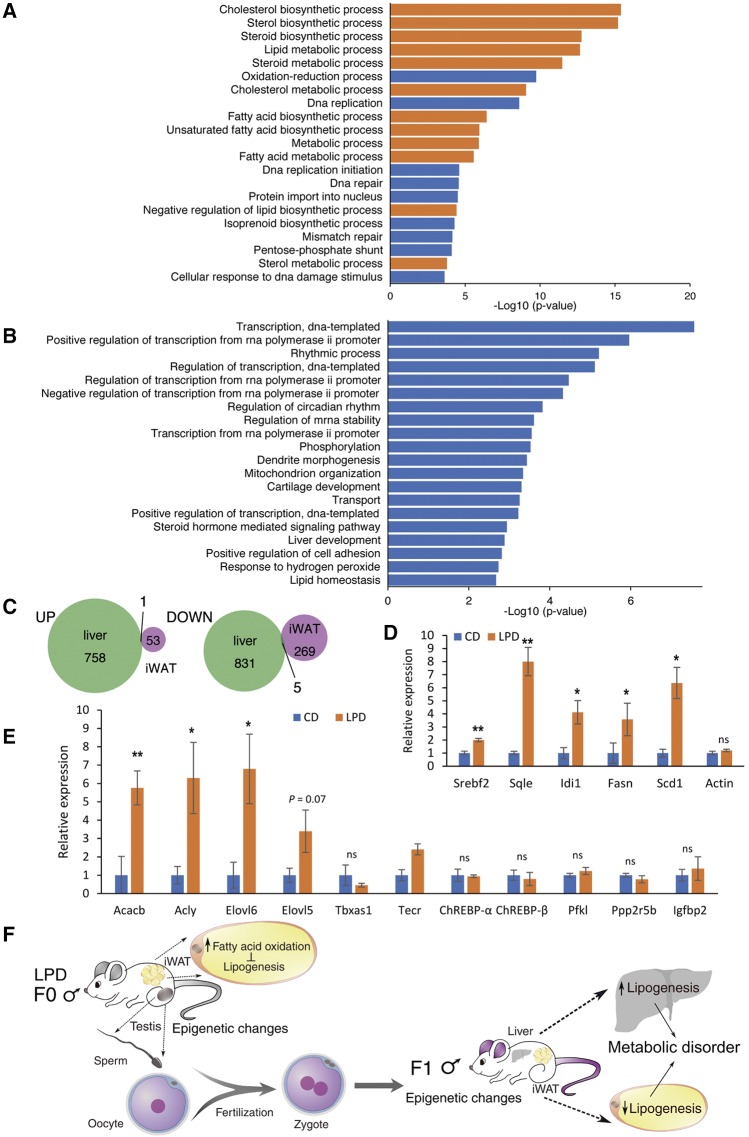
Comparison of pLPD-regulated genes in iWAT and liver of F1 offspring mice. (A and B) The up- and down-regulated genes previously reported by Carone *et al.* in the liver of F1 offspring (Carone *et al.* 2010) were used for this analysis. Gene ontology analysis for biological processes (GO-BP) for up- (A) and down-regulated (B) genes, respectively, was performed as described in Figure 1A. Only top 21 terms for upregulated genes and 20 terms for downregulated genes are shown. Genes involved in cholesterol biosynthesis and fatty acid metabolism are enriched among the up-regulated genes, and shown in orange. (C) Comparison of the pLPD-regulated genes in the liver and iWAT of F1 offspring. The Venn diagram shows the numbers of up- and down-regulated genes in the liver, as reported by Carone *et al.* (Carone *et al.* 2010), and in the iWAT (shown in Figure 1) of F1 offspring. (D) Expression levels of genes related to lipid and cholesterol biosynthesis in the offspring liver measured by qRT-PCR were consistent with the previous study (Carone *et al.*, 2010) (n = 3). (E) Validation of expression levels of genes of interest in iWAT in the offspring liver (n = 3). (For D and E) Expression level was normalized to the reference gene 36B4, and actin was used as a negative control. Bars represent the mean ± SD *, *P* < 0.05; **, *P* < 0.01; ns, not significant; CD, control diet; LPD, low-protein diet. (F) Model depicting how the gene expression changes induced by pLPD feeding in iWAT and liver could cause metabolic disorders in offspring.

To understand how pLPD-induced differences in gene expression in the iWAT and liver of the offspring contribute to the glucose intolerance that was previously reported (Watkins and Sinclair 2014), we also determined the DEGs in F1 liver, which was previously analyzed following a similar feeding study (Carone *et al.* 2010). We again performed pathway analysis of the up- and down- regulated genes. In liver, of the 1,559 DEGs (FDR < 0.001), 759 genes were up-regulated and 836 genes were down-regulated. KEGG pathway analysis identified the pathways that were enriched among the up- and down-regulated genes, respectively (Figure 4A and 4B). Of the top 21 pathways represented among the up-regulated genes, 10 are involved in cholesterol biosynthesis and lipid metabolism, whereas none of the pathways represented among the down-regulated genes are related to lipid metabolism. Comparison of the DEGs in F1 iWAT and liver showed almost no overlap, with only one and five genes overlapping among the up- and down-regulated lists, respectively (Figure 4C), and none of these are involved in metabolism. To confirm that the result of Carone *et al.* (2010) are reproducibly obtained in the present study, we performed qRT-PCR to examine the expression levels of typical genes involved in lipid and cholesterol biosynthesis, including sterol regulatory element binding transcription factor 2 (*Srebf2*), squalene spoxidase (*Sqle*), isopentenyl-diphosphate delta-isomerase 1 (*Idi1*), stearoyl-CoA desaturase (*Scd1*) and fatty acid synthase (*Fasn*). As shown in Figure 4D, our results were consistent with previous study (Carone *et al.* 2010), and indicated that the expression levels of these lipogenic genes were significantly increased by pLPD feeding. The up-regulation of many genes involved in lipid and cholesterol biosynthesis suggests that lipogenesis may be up-regulated by pLPD feeding in F1 liver. To assess whether the lipogenic genes and regulators which were differentially expressed in iWAT are oppositely expressed in liver, we measured the expression of these genes in the livers of the offspring as in Figure 4E. Except for *Tbxas1*, expression levels of *Acacb*, *Acly*, *Elovl5*, *Elovl6* and *Tecr* that were reduced in iWAT were strongly increased in pLPD offspring liver compared to CD mice, which was also consistent with previous study, indicating the opposite expression of lipogenic genes in liver and iWAT. The expressions of *ChREBP*-*α*, *ChREBP*-*β*, *Pfkl*, *Ppp2r5b* and *Igfbp2* were unchanged, suggesting that ChREBP did not contribute to the increase of lipogenesis in the liver. Instead, we found the up-regulation of lipogenic transcriptional factor *Srebf2* (Figure 4D). These results support the previous study showing that up-regulation of hepatic lipogenesis is mediated by *Srebf2* (Carone *et al.* 2010). Also, high expression of genes related to lipogenesis in pLPD offspring liver can be due to higher expression of SREBP-activating gene *Scap* (Carone *et al.* 2010). The findings here are in agreement with the fact that SREBP is the key regulator of lipogenesis in liver, whereas ChREPB plays dominant roles in regulation of lipogenesis in adipose tissue (Horton *et al.* 2002, Herman *et al.* 2012). Threfore, our results demonstrate that lipogenic genes are reciprocally expressed in iWAT and liver in the pLPD offspring, and their expressions are regulated by different transcriptional factors in a tissue-specific manner. It has previously been shown that lipogenesis in WAT and liver oppositely correlate with insulin resistance: a lower lipogenic rate in WAT and a higher lipogenic rate in liver cause insulin resistance (Cao *et al.* 2008; Yang *et al.* 2011; Huo *et al.* 2012; Chavez and Summers 2012; Beaven *et al.* 2013; Abdul-Wahed *et al.* 2017; Vijayakumar *et al.* 2017). Taken together, our results therefore suggest that disorders of lipid metabolism in liver and white adipose tissue in juvenile mice of LPD fathers may play pivotal roles in the development of metabolic disorders (Figure 4F).

## Discussion

The present study indicates that pLPD down-regulates multiple lipogenic genes in offspring iWAT, which may reduce lipogenesis and increase the risk of metabolic disorders. Down-regulation of *ChREBP*-*β*, a key transcription factor for lipogenic genes, which might be the result of an increase in *Igfbp2* expression, is likely to play an important role in this down-regulation. We also identified pLPD-induced up-regulation of genes involved in the immune response, including pellino-1 (*Peli1*) and high mobility group box 2 (*Hmgb2*), in offspring iWAT. It was previously shown that inflammation prevents adipocyte differentiation by inhibiting PPARα and PPARγ (Rosen & MacDougald 2006). However, there is no clear evidence to show that some of these up-regulated genes enhance inflammation, although some reports suggest the possibility that PELI1 and HMGB2 may regulate inflammation (Aird *et al.* 2016; Kim *et al.* 2017). Thus, up-regulation of immune response-related genes may not directly contribute to the down-regulation of lipogenic genes by inhibiting adipocyte differentiation.

Many studies have shown an effect of pLPD on offspring gene expression in rodents, but comparison of the data in the reports is not always possible because of the use of different diets and feeding regimens. To enable comparison of our data with those in the previous report by Carone *et al.* (Carone *et al.* 2010), we used the same diet and feeding regimen. However, there still exist differences between two studies. First, 3-week-old mice were used in the study by Carone *et al.* (2010). In this study, because iWAT of 3-week-old-mice were quite small and contains high amount of lipid which affects the efficiency of RNA extraction, we used the 5-week-old offspring in which the offspring are still young and their adipose tissues are also sufficient for experimental manipulation. Second, C57BL/6 mice in Carone *et al.* (2010) were obtained from Jackson Labs and from Charles River Laboratories, whereas our C57BL/6 mice were obtained from Shizuoka, Japan. These mice are substrains of B6 mice. Despite these differences, in the offspring liver, we also found the up-regulation of genes relating to lipid and cholesterol biosynthesis, which was consistent with Carone *et al.* (2010). This data suggested that lipid metabolism is sensitive to pLPD in spite of some of genetic background differences among B6 substrains (Mekada *et al.* 2009). The Technology and Development Team for Mouse Phenotype Analysis of RIKEN BRC also confirmed that there is no significant difference in the glucose tolerance test between C57BL/6 mice from SLC and Charles River Laboratories (data not shown). In contrast to liver, we found that lipogenic genes were reduced in iWAT, which was partly due to the reduction of *ChREBP*-*β*. It was demonstrated that loss of adipose-ChREBP results in insulin resistance and increased WAT inflammation (Vijayakumar *et al.* 2017). In our RNA-seq data, we noted that mRNA level of *TNF*-*α* gene in the iWAT had a tendency to increase, albeit at the border of significance (log_2_FC = 2.18, FDR = 0.07, data not shown), suggesting a low-grade inflammation in adipose tissue of pLPD offspring. Previously, Watkins and Sinclair (2014) reported that the adult offspring from LPD-fed fathers developed impaired glucose tolerance and increased serum TNF-α level (Watkins and Sinclair 2014). Our results could imply that lipid metabolic disorders in liver and white adipose tissue during early life contribute to the development of such diseases in these mice. However, between our study and the study by Watkins and Sinclair (2014), there are many other differences in experimental components, including protein concentration, feeding scheme, and age at mating, in addition to the difference of C57/BL6 genetic background. Nevertheless, the important factors that are likely to contribute to the effect of pLPD may include protein concentration and exposure time. In both studies, protein amount was reduced by about half (11% and 9% in this study and Watkins & Sinclair study, respectively); more importantly, the time of LPD exposure of at least 7 weeks in two studies was assured for the exposure of LPD during a complete cycle of male germ cell formation. Therefore, we speculate that although the adult offspring in our study may also develop metabolic syndrome, but the types of disease and/or the degree of progress of disease may differ from study of Watkins and Sinclair (2014). More studies are needed to investigate the traits during the development of the pLPD offspring. Overall, our findings based on enriched genes and pathways suggest that lipid metabolism was disturbed in liver and white adipose tissue in the young offspring from LPD fathers, which may coordinately increase risk of metabolic disorders.

Almost no overlap was found between the DEGs in iWAT and liver, and it is as yet unclear how a pLPD might differentially affect the expression of genes in these two offspring tissues. LPD may induce an epigenetic change, such as histone modification or RNA induction, in the testicular germ cells (TGCs), which may be transmitted to the mature sperm. Although it has been shown that LPD does not affect DNA methylation pattern in mature sperm, it is unknown whether LPD can cause histone modification or alter RNA content in mature sperm (Shea *et al.* 2015). Specific histone modifications and/or RNAs in sperm may be transmitted to the zygote, which could induce an epigenetic change and/or alter gene expression in the early embryo. Because differentiation into tissues such as liver and WAT occurs in the post-blastocyst embryo, such changes in the early embryo would be expected to affect gene expression in the embryo at a later stage. Thus, further analysis is required to dissect the mechanism underlying the differential effects of a pLPD on the expression of genes in iWAT and liver.

The molecular mechanism underlying the transmission of pLPD effects is not fully understood. It has also been recently reported that sperm from mice fed a LPD and those fed a high-fat diet contain higher concentrations of tRNA fragments (Chen *et al.* 2016; Sharma *et al.* 2016), and that injection of sperm small RNAs from HFD-fed mice into zygotes causes metabolic disorders and alters the expression of genes in metabolic pathways in the offspring (Chen *et al.* 2016). However, the type of epigenetic change induced by LPD in TGCs and mature sperm remain to be established. Our previous studies showed that, in response to various environmental factors, including heat shock, psychological stress, inflammatory cytokines, and pathogen infection, the stress-responsive epigenetic regulator *Drosophila* ATF2 (dATF2) and the vertebrate ortholog ATF7 are phosphorylated by p38 and released from the chromatin, resulting in a reduction in histone H3K9 tri- and di-methylation (H3K9me3/2), which can be transmitted to the subsequent generation in some instances (Maekawa *et al.* 2010; Seong *et al.* 2011; Seong *et al.* 2012; Yoshida *et al.* 2015). We hypothesize that ATF7 may play role in this transmission because it is reported that LPD feeding reduces the concentration of glutathione (GST) (Leaf and Neyberger 1947), an abundant cysteine-containing tripeptide that removes ROS (Hayes and McLellan 1999), which activate p38 (Ito *et al.* 2006). Hence, it is possible that LPD induces phosphorylation of ATF7 via p38, finally leading to a change of histone H3K9me3/2, as previously reported (Maekawa *et al.* 2010; Seong *et al.* 2011; Seong *et al.* 2012; Yoshida *et al.* 2015). In addition, epigenetic changes may be induced by the inhibition of α-ketoglutarate-dependent dioxygenases that are involved in DNA and histone demethylation, secondary to altered levels of fumarate and succinate, key metabolites in the TCA cycle (Xiao *et al.* 2012; Sciacovelli *et al.* 2016). Future experiments are required to dissect the mechanism underlying the inheritance of pLPD effects.

A LPD up-regulates the expression of lipid oxidation genes in the iWAT of F0 mice, but down-regulates lipogenic gene expression in offspring iWAT. Differences in gene expression in offspring WAT may be the result of epigenetic changes in the TGCs of their male progenitors. Although epigenetic changes may be similar across all types of cells, including iWAT and TGCs, epigenetic changes appear to be induced on different genes by LPD in iWAT and TGCs. This may be due to differences in chromatin structure, such as differences in heterochromatin in each cell type. In addition to pericentromeric and subtelomeric heterochromatin, many genes in the euchromatin region also have heterochromatin-like structures enriched in H3K9me3/2, and this differs among cell types. Therefore, LPD may induce epigenetic changes on different genes depending on cell type, despite similar mechanisms of epigenetic modification.

## Conclusions

Many studies have shown that parental diet affects the metabolism and health of their offspring (Barker *et al.* 1993; Anderson *et al.* 2006; Ng *et al.* 2010; Carone *et al.* 2010; Radford *et al.* 2014; Watkins and Sinclair 2014). To understand the relationship between parental diet and offspring health, tissue-specific analysis of gene expression changes induced by paternal dietary manipulation is essential. The present study of gene expression in iWAT and liver may provide useful insight into the molecular mechanisms underlying the effect of a pLPD on gene expression patterns in the offspring.
